# Single-center versus multi-center data sets for molecular prognostic modeling: a simulation study

**DOI:** 10.1186/s13014-020-01543-1

**Published:** 2020-05-14

**Authors:** Daniel Samaga, Roman Hornung, Herbert Braselmann, Julia Hess, Horst Zitzelsberger, Claus Belka, Anne-Laure Boulesteix, Kristian Unger

**Affiliations:** 1 Helmholtz Zentrum, München, Ingolstädter Landstr. 1, Neuherberg, 85764 Germany; 2 Department of Medical Information Processing, Biometry and Epidemiology, University of Munich, Marchioninistr. 15, Munich, 81377 Germany; 3 Clinical Cooperation Group Personalized Radiotherapy in Head and Neck Cancer, Helmholtz Zentrum München, Research Center for Environmental Health (GmbH), Munich, Ingolstädter Landstr. 1, Munich, 85764 Germany; 4 Department of Radiation Oncology, University Hospital, LMU Munich, Marchioninistr. 15, Munich, 81377 Germany

**Keywords:** Predictive model, Omics data, Feature selection, Predictive performance, Study design, Validation

## Abstract

**Background:**

Prognostic models based on high-dimensional omics data generated from clinical patient samples, such as tumor tissues or biopsies, are increasingly used for prognosis of radio-therapeutic success. The model development process requires two independent discovery and validation data sets. Each of them may contain samples collected in a single center or a collection of samples from multiple centers. Multi-center data tend to be more heterogeneous than single-center data but are less affected by potential site-specific biases. Optimal use of limited data resources for discovery and validation with respect to the expected success of a study requires dispassionate, objective decision-making. In this work, we addressed the impact of the choice of single-center and multi-center data as discovery and validation data sets, and assessed how this impact depends on the three data characteristics signal strength, number of informative features and sample size.

**Methods:**

We set up a simulation study to quantify the predictive performance of a model trained and validated on different combinations of in silico single-center and multi-center data. The standard bioinformatical analysis workflow of batch correction, feature selection and parameter estimation was emulated. For the determination of model quality, four measures were used: false discovery rate, prediction error, chance of successful validation (significant correlation of predicted and true validation data outcome) and model calibration.

**Results:**

In agreement with literature about generalizability of signatures, prognostic models fitted to multi-center data consistently outperformed their single-center counterparts when the prediction error was the quality criterion of interest. However, for low signal strengths and small sample sizes, single-center discovery sets showed superior performance with respect to false discovery rate and chance of successful validation.

**Conclusions:**

With regard to decision making, this simulation study underlines the importance of study aims being defined precisely a priori. Minimization of the prediction error requires multi-center discovery data, whereas single-center data are preferable with respect to false discovery rate and chance of successful validation when the expected signal or sample size is low. In contrast, the choice of validation data solely affects the quality of the estimator of the prediction error, which was more precise on multi-center validation data.

## Background

Oncological treatment is based on surgery, radiotherapy, chemotherapy and immunotherapy for reduction of tumor burden and for improvement of local control of the tumor. Of particular importance is radiotherapy, which has been shown in numerous studies to improve local control and overall survival of patients [1, 2]. Radiation oncology treatment strives to optimize the reduction of tumor cells while preserving the surrounding non-tumor tissue. Effectiveness is influenced by a number of factors such as radiation sensitivity, the anatomical borders and immunogenic constitution of the tumor, and its environment [1]. The interplay between these factors is complex and prediction of the radiation response and overall clinical performance requires detailed measurement of the underlying molecular state of the tissue. This is increasingly attempted through the use of systemic multi-level omics biology approaches [3, 4]. The complexity of the interplay is consistently reflected in the heterogeneous risks of subgroups of cancer patients in terms of local and distant control and overall survival, e.g. in head and neck cancer or glioblastoma [5, 6]. This heterogeneity is a great challenge in oncology since it means that only a subgroup of treated patients is likely to benefit from standard therapy. Hence, the need for prognostic factors predicting individual response is great and a lot of research effort has been invested in the past decade to identify molecular prognostic markers from multi-level omics data generated from clinical patient samples. Examples that have reached clinical practice are the diagnostic assays OncotypeDX and Mammaprint, which predict the risk of recurrence or metastasis in breast cancer [7, 8]. For locally advanced head and neck cancer and glioblastoma, prognostic gene and miRNA signatures predicting local and distant control or overall survival have been recently identified and are promising markers with the potential to allow substratification of standard-therapy treated patients for alternative treatment strategies [9–11].

From a methodological point of view, molecular prognostic models are specialized statistical regression models that generate signatures from molecular data measured in biological samples such as peripheral blood, resected tumor tissue or tumor biopsies. A major task in prognostic modeling using high-dimensional molecular data is feature selection, which is often realized by the least absolute shrinkage and selection operator, called the Lasso [12]. The selected features with non-zero estimated coefficients in the prognostic model form a so-called signature. Conceptually, the approach of using molecular information for prognostic modeling is backed by the finding that many cancer types are tremendously heterogeneous and form subgroups of different prognosis or different therapeutic accessibility [13–15]. Consequently, high-dimensional measurements at the genome, transcriptome, post-transcriptome and protein levels, individually or in combination, were used to generate signatures for the stratification of breast carcinomas [13, 16–20], glioblastoma [11, 21], gastric cancer [22, 23], lung adenocarcinomas [24], squamous cell cervical carcinoma [25] and head and neck squamous cell carcinomas [10, 26, 27].

For all statistical models, the “predictive accuracy on test sets is the criterion for how good the model is" [28]. In other words, for prognosis the “usefulness is determined by how well a model works in practice, not by how many zeros there are in the associated *P*-values" [29]. Thus, with respect to radiotherapy, the signature must predict satisfactorily well the treatment outcome of patients other than those the model was developed on. For prognostic models in the clinical context, external validation is commonly considered as the most relevant form of validation [29]. Studies aiming at new prognostic signatures therefore require two independent cohorts; the discovery cohort is used to identify a signature from the high-dimensional data and the validation cohort is used to measure its performance. Note that split sample approaches (including cross-validation with leave-one-out cross-validation as a special case) are a form of internal validation and therefore are structurally insufficient for estimating the generalization performance of signatures; instead, external validation is required. Systematic reviews retrospectively enlighten the quality of validation strategies and indicate potential lacks of thoroughness if present [30].

Collecting data sets suitable for molecular prognostic modeling is a tedious task for several reasons. Firstly, the number of patients that are homogeneous with respect to cancer subtype and clinical factors is very limited in most clinical sites. Secondly, each clinical sample is generated from tumor tissue, biopsies or blood samples of a patient. As a consequence, data sets of sufficient size either come from a large single clinical site (single-center (SC) data, e.g. Clinical Cooperation Group [5]), are collected from multiple clinical sites (multi-center (MC) data, e.g. German Cancer Consortium [31]) or are taken from large databases (MC, e.g. The Cancer Genome Atlas [15]). Even if cases are assumed to be homogeneous across centers, there is evidence that site-specific factors influence molecular high-throughput data despite all standardization efforts being made across clinical sites [32–34]. Therefore, SC data is more homogeneous, whereas, as a general hypothesis, MC data shows better generalizability. Moreover, it has been observed that SC studies are overoptimistic in terms of estimated effect sizes [35]. Furthermore, center-heterogeneity is sometimes viewed as a potential reason for failed validation in mono-institutional validation studies [36].

Shared noise patterns among samples, independent of the biological factor of interest, are called batch effects and mask information. They occur particularly with complex measurement techniques that process many probes at a time; in microarray experiments, samples being processed on the same multiwell plate form batches that share various noise patterns [37]. Therefore, center effects are structurally a mixture of batch effects and case mix effects, the latter describing effects caused by differences regarding the case-composition of the center-wise patient cohorts. Since batch effects occur regularly in microarray-based studies, strategies for batch correction are well analyzed and discussed with respect to sample size and effect size [37–40]. The dominant strategies for batch correction are methods of location and scale adjustment or matrix factorization [38]. Although batch correction can mitigate the deranging influence, no method can spirit away the effect completely. Thus, prognostic models for tumor samples have to deal with the batch patterns of the clinical centers involved.

For prognostic modeling, two data sets are required. When a SC and a MC data set are available, this raises questions about how to make best use of the data. Which data should be used for discovery and which for validation? Both strategies (i.e. using the SC data set for discovery and MC data set for validation, or the other way around) have been applied recently for prognostic modeling of radiotherapy treatment outcomes using molecular data [10, 11]. More generally, the question arises about whether a researcher should aim for SC or MC data, when using resources for data acquisition.

In this article, we address decision making regarding the choice of SC or MC data for discovery and validation cohorts for prognostic modeling–as this is often needed in studies for predicting the outcome of radiotherapy from high-dimensional molecular data. In addition to the scenario where an SC and an MC data set are available and have to be assigned to either discovery or validation, we also consider two scenarios in which only SC or only MC data are used for both discovery and validation. We use the Hornung model to simulate gene expression data sets representing different centers affected by batch effects [39]. We vary the model parameters signal strength, number of informative genes, and sample size and show their impact on the best choice.

To our knowledge, there is no systematic study that investigates the performance of feature selection procedures of regression models in the presence of batch effects. We present a study based on simulated gene expression data that focuses on batch-type center-effect while ignoring case mix effect.

## Methods

The heterogeneity of microarray data from different clinical sites was modelled by the Hornung batch model [39]. We chose to focus on the multiple linear regression model to avoid problems related censored survival times and estimation of the baseline hazard function, which would only distract from the actual issue of interest and potentially blur the simulation results. Our simulation study compares the four possible combinations of SC and MC data for discovery and validation of prognostic models. The parameters (i) signal strength, (ii) number of informative genes and (iii) sample size were systematically varied in three separate scenarios.

**Fig. 1:**
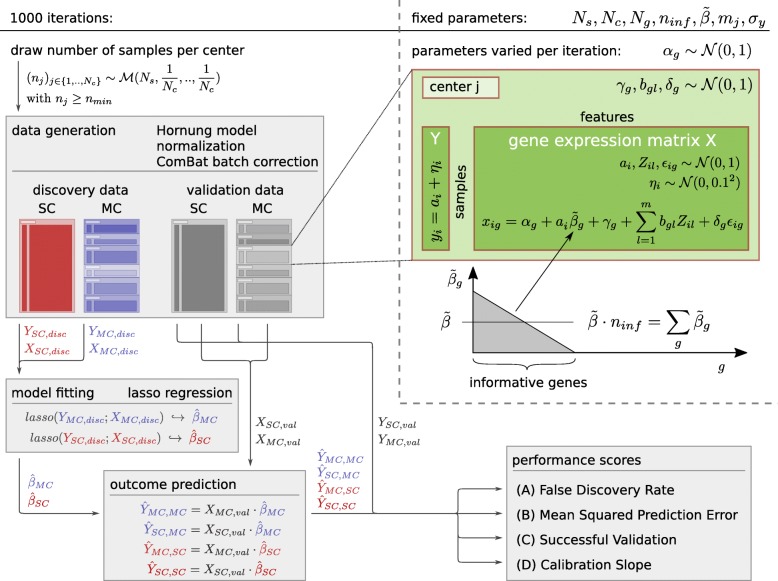
Simulation scheme for the computation of performance scores of molecular prognostic models with center effects. For each parameter set 1000 Monte-Carlo runs are performed. In single-center (SC) and multi-center (MC) data sets, independent variables *X* and dependent variables *Y* are generated from a true state *a*, noise and the center batch pattern. Each center shares realizations of randomly sampled batch-specific parameters among its samples. MC data sets are batch corrected, a signature is fitted to the discovery data and used for prediction of validation data. Performance scores are calculated to measure the average quality of prediction

In each scenario, the comparison of the four data set combinations – (SC discovery, SC validation), (SC,MC), (MC,SC) and (MC,MC) – is based on four performance scores that were calculated from 1000 iterations (per parameter set) of data generation, model fitting and validation. Considering the (small) width of the confidence intervals of the simulation results (see “Results” section), this number was considered to be a good compromise between computing time and precision. In each realization we first generated high-dimensional data matrices for SC and MC discovery and validation data according to the Hornung model. This means that for sample *i* of center *j* a true state *a*_*ij*_, an observed state *y*_*ij*_ as well as for every gene *g* an expression value *x*_*ijg*_ were calculated, the latter being composed of the signal (i.e. expression levels caused by the true state), a center-specific batch pattern and noise. After normalization and batch effect correction using ComBat, we then regressed the observed state vector on the gene expression matrix using the Lasso method and obtained candidate signatures for the SC and MC discovery data. This means that the observed states are used as dependent variable in the lasso regression and the true states can be seen as this variable without measurement error. The candidate signatures were then applied to the gene expression matrices of the validation data in order to predict the corresponding observed states in the validation data. Finally, we calculated the performance scores from the deviations of the predicted from the observed states of the validation data. An overview of the simulation scheme is given in Fig. 1. Case mix effects and similar sources of heterogeneity, like batch-wise varying signal strength, were not considered.

### Generation of data by Hornung model

Hornung et al. [39] presented a model to generate data affected by batch effects by setting the measured expression level of gene *g* for sample *i* of batch *j* to:

$$x_{ijg} = \alpha_{g} + a_{ij} \tilde{\beta}_{g} + \gamma_{jg} + \sum_{l=1}^{m_{j}} b_{jgl}Z_{ijl} + \delta_{jg} \epsilon_{ijg}. $$

Thus, each data point *x*_*ijg*_ is constructed as the sum of a basal gene level *α*_*g*_, the product of the effect size $\tilde {\beta }_{g}$ and the individual true state *a*_*ij*_ (representing the signal for sample *i* from batch *j* in gene *g*), a batch-specific shift on each gene *γ*_*jg*_, the weighted sum of *m*_*j*_ random latent factors with coefficients *b*_*jgl*_ (representing unobserved environmental, demographic and technical factors [41] that introduce center-wise correlation patterns among the features and are uncorrelated to the true state) and individual weights *Z*_*ijl*_, and the product of noise *ε*_*ijg*_ and a batch- and gene specific scaling factor *δ*_*jg*_. In contrast to the original use of the model for binary target variables, we used continuous true states *a*_*ij*_ that were measured with additive noise, such that the target was modelled as *y*_*ij*_=*a*_*ij*_+*η*_*ij*_, with $\eta _{ij} \sim \mathcal {N}(0,\sigma _{y}^{2})$. Note that, in contrast to the commonly considered modeling of a target variable *y* (representing the outcome in a multiple linear regression) as a noisy function of the multidimensional variable *x*, in the Hornung model, *x* and *y* are both modelled as functions of an unobserved true state *a*.

#### Default parameter settings

Unless specified differently, *N*_*s*_=100 samples were generated per data set (*i*∈{1,..,*N*_*s*_}). For MC settings, the samples were distributed randomly to *N*_*c*_=8 centers (*j*∈{1,..,*N*_*c*_}) [10]. Thereby, the number of samples was constrained by a minimum of *n*_*min*_=10 samples per center (realized by assigning *n*_*min*_ samples to all centers and subsequently distributing the remaining samples with equal probability).

In our simulations, we chose normally distributed gene wise basal expression levels ($\alpha _{g} \sim \mathcal {N}(0,1)$). The true state was also chosen to be normally distributed ($a_{ij} \sim \mathcal {N}(0,1)$). The target variable *y*_*ij*_ was modelled as a realization of the true state *a*_*ij*_ distorted by additive noise with a standard deviation of 0.1:

$$y_{ij} = a_{ij} + \eta_{ij}, \hspace{0.25cm} \eta_{ij} \sim \mathcal{N}(0,0.1^{2}). $$

Following Hornung et al. [39], samples consist of *N*_*g*_=1000 genes (*g*∈{1,..,*N*_*g*_}). A fraction of 30% of the genes was considered to be informative (*n*_*inf*_=0.3·*N*_*g*_), which means—without loss of generality, that $\tilde {\beta }_{g} \neq 0$ for *g*≤*n*_*inf*_ [39]. The effect size for an informative gene *g* was chosen to be graded linearly with average $\tilde {\beta }$ as $\tilde {\beta }_{g} = 2 \tilde {\beta } \left (1 - \frac {g}{n_{inf}+1}\right)$, non-informative genes were given an effect size of zero. Batch specific shifts were normally distributed ($\gamma _{jg} \sim \mathcal {N}(0,1)$). Following Hornung et al., we used *m*_*j*_=5 latent factors [39]. We drew the coefficients as $b_{jgl} \sim \mathcal {N}(0,1)$. Noise terms *δ*_*jg*_ and *ε*_*ijg*_ were also normally distributed with mean 0 and variance 1. In each run, for every center a unique batch pattern was generated following the model with the parameters specified in Table 1.

**Table 1:** Parameters of simulations

			Sc1	Sc2	Sc3
signal strength	$\tilde {\beta }$	=	[0; 0.5]	0.25	0.125
number of genes, informative	*n*_*inf*_	=	300	[1;1000]	300
sample size	*N*_*s*_	=	100	100	[40 500]
number of genes, total	*N*_*g*_	=	10^3^	10^3^	10^3^
number of centers in MC	*N*_*c*_	=	8	8	8
minimum samples per center	*n*_*min*_	=	10	10	5
basal level gene *g*	*α*_*g*_	∼	$\mathcal {N}(0,1)$	$\mathcal {N}(0,1)$	$\mathcal {N}(0,1)$
target	*a*_*ij*_	∼	$\mathcal {N}(0,1)$	$\mathcal {N}(0,1)$	$\mathcal {N}(0,1)$
fixed batch effect gene *g*	*γ*_*jg*_	∼	$\mathcal {N}(0,1)$	$\mathcal {N}(0,1)$	$\mathcal {N}(0,1)$
number of latent factors	*m*_*j*_	=	5	5	5
factor loadings	*b*_*jgl*_	∼	$\mathcal {N}(0,1)$	$\mathcal {N}(0,1)$	$\mathcal {N}(0,1)$
impact of factor *l* on sample *i*	*Z*_*ijl*_	∼	$\mathcal {N}(0,1)$	$\mathcal {N}(0,1)$	$\mathcal {N}(0,1)$
noise scaling of gene *g* in batch *j*	*δ*_*jg*_	∼	$\mathcal {N}(0,1)$	$\mathcal {N}(0,1)$	$\mathcal {N}(0,1)$
noise	*ε*_*ijg*_	∼	$\mathcal {N}(0,1)$	$\mathcal {N}(0,1)$	$\mathcal {N}(0,1)$
standard deviation of observation noise	*σ*_*y*_	=	0.1	0.1	0.1

Each column shows the parameter set for one of three simulated scenarios. The intervals indicate the ranges in which the parameter values were varied in the respective scenarios. Fixed parameters are indicated by ‘ =’, while sources of heterogeneity as signal, noise and batch effects are characterized by the parameters of their densities, indicated by the ’ ∼’ symbol

### Scenarios: systematic parameter variation

The main goal of the simulation study was to investigate the influences of three different factors (signal strength, number of informative genes and sample size) on the performances measured when using SC data and MC data for discovery and validation. To this end, three different scenarios were considered; in each of these a single factor was varied systematically to investigate its influence and discern this from the influences of the other two factors. For each of the three scenarios, the values of the parameters not explicitly mentioned in the following descriptions were fixed to the values given in “Default parameter settings” section.

#### (i) signal strength

Taking the variable *a*_*ij*_ as the biological true state, the parameter $\tilde {\beta }_{g}$ defines the impact of the true state on the measured expression level *x*_*ijg*_. Thus, larger values of $\tilde {\beta }_{g}$ increase the signal in the covariates, without changing the variance of the outcome variable across simulations. Strictly speaking, this parameter describes to what extent a true state *a*_*ij*_ influences the measured expression level *x*_*ijg*_, which reflects the effectiveness. For the sake of simplicity, we denote it as signal strength. In this scenario, $\tilde {\beta }$ was stepwise increased from 0 to 0.5 (taking the values {0, 1,.., 9 }/50, {8, 9,.., 15 }/40, {40, 43, 46, 50 }/100). The number of informative genes was kept constant at 300, which is the same value used by Hornung et al. [39]. The sample size was set to 100, which corresponds to the order of magnitude of the MC and SC data of Hess et al. [10].

#### (ii) number of informative features

In the second scenario, the impact of the signal spikiness was analysed. The performance of prognostic models using SC and/or MC data consisting of 1000 features with only a few features carrying strong signals was contrasted to the performance in the case of many informative features carrying weak signals. Taking $\iota = \sum _{g=1}^{n_{inf}} |\tilde {\beta }_{g}|$ as a measure for information in the data, we kept *ι* constant throughout all settings of this scenario and varied $\tilde {\beta }_{g}$ as a function of *n*_*inf*_. For the default number of 300 informative features, we chose a signal strength of $\tilde {\beta } = 0.25$, which is just the middle of the covered parameter interval of scenario (i). The information value *ι* was kept constant at 300·0.25 over all settings and the signal strength parameter value in each setting was calculated according to the respective number of informative features. The number of informative features *n*_*inf*_ was varied from 1 to 1000 (taking the values 1, 5, 10, 25, 50, 75, 100, 150, 200, 250, 300, 350, 400, 450, 500, 550, 600, 670, 750, 850, and 1000). The sample size was again set to 100, in order to match the first scenario.

#### (iii) sample size

The sample size obviously plays a major role in identifying signals in noisy settings. Therefore, the sample size was varied from 40 to 500 (taking values 40, 45, 50, 60, 80, 100, 125, 150, 200, 250, 350, and 500). In this scenario, the minimum number of samples per center *n*_*min*_ was reduced to 5 in order to allow for sample sizes as low as 40. The number of informative features was again set to the default value of *n*_*inf*_=300, whereas the signal strength was reduced to $\tilde {\beta } = 0.125$ in order to prevent unrealistically strong signals in the cases of the larger sample sizes.

### Normalization and batch correction

Initially every generated sample was normalized to have zero mean and unit standard deviation. After normalization, MC data sets were batch corrected using standard tool ComBat [37]. For all following analyses, readily processed (normalized and batch-corrected) discovery and validation data are denoted as *X*_*c*,*disc*_ and *X*_*c*,*val*_, respectively, with *c*∈{*MC*,*SC*} indicating whether data was generated from one or more centers.

### Model fitting

To identify a signature $\hat {\beta }_{c}$, a linear model

$$Y_{c,disc} = \beta_ 0 + X_{c,disc}\beta + \varepsilon; \hspace{0.25cm} \varepsilon \sim \mathcal{N}(0,\sigma_{e}^{2}) $$

was fitted to the discovery data using the Lasso method [12], as implemented in the R package glmnet (cv.glmnet function) [42]. In Lasso regression, the criterion to be minimized is the sum of squared errors *plus* a penalty term that penalizes the absolute values of *β*. By constraining the coefficients in this way, some coefficients (hopefully those of non-informative genes) are pushed to zero and the remaining genes—with non-zero coefficients— are considered selected and form the signature defining the prognostic model. Lasso regression involves a tuning parameter called *λ* that has to be chosen. A common approach, implemented in the function predict.cv.glmnet (through the option ‘s = lambda.1se’) and adopted here, is to use a slightly more strongly penalizing *λ* value than the one obtained from minimizing the cross-validated prediction error.

### Performance scores

Four performance scores were calculated from 1000 runs (*N*_*sim*_=10^3^). The mean values of the four scores and the corresponding standard errors of the mean are reported. For visualization, the mean values and the corresponding 99% confidence intervals are plotted.

We included the results obtained for all simulation iterations in the evaluation. Thus, we also included the iterations in which lasso did not select any variable, even though it would not be meaningful to use empty signatures in practice. Excluding these iterations would have potentially biased the results; it is important to keep the evaluation of simulation studies neutral by considering each simulation iteration instead of letting the results influence the decision on whether or not to include the individual iterations. Nevertheless, we also analyzed the performance of non-empty signatures separately and the results did not change substantially (data not shown).

The 99% confidence intervals contain the true means with probability 0.99. Thus, non-overlapping confidence bands are a strong indication for systematic differences between the data usage settings.

Performance scores were calculated by the following procedure. The two signatures $\hat {\beta }_{MC}$ and $\hat {\beta }_{SC}$ were used to predict the target variable *Y*_*p*,*val*_ of the independent validation data sets from their expression data *X*_*p*,*val*_ by

$$\hat{Y}_{p,c} = \hat{\beta}_{0} + X_{p,val}\hat{\beta}_{c}; \hspace{0.5cm} p,c \in \{MC, SC\}. $$

Four performance scores were computed in every iteration based on the estimated signature $\hat {\beta }_{c}$ and on the deviation of the prediction $\hat {Y}_{p,c}$ from the true values *Y*_*p*,*val*_ of the target variable in the validation data.

#### (A) false discovery rate: FDR

Usefulness of a signature is connected with the identification of informative features. Particularly, any element of the gene set returned by model fitting should be unlikely to be a false positive finding.

The FDR returns the proportion of features in a signature, that are actually non-informative.

In empty signatures, this proportion does not exist. The FDR of empty signatures was set to 1. The rationale for assigning the worst score is that in all simulations (with $\tilde {\beta } \neq 0$) truly was signal in the data, which was completely missed by the model fitting in those realizations. Missing all existing information in prognostic modeling is a clear failure and far from the goal of signatures built of informative features.

#### (B) mean square prediction error: MSPE

The most common and most important performance score is the expected prediction error. Particularly for clinical applications, the prognosis should be as close to the true outcome as reasonably achievable.

The MSPE of a signature in a validation data set is an estimator for the expected squared prediction error of single future samples. Because batch correction is not possible in single sample prediction, the batch correction of the validation data is removed for calculating the MSPE (in contrast to the other performance scores). The MSPE is defined as

$$MSPE_{p,c} = \frac{1}{N_{s}} \sum_{i=1}^{N_{s}}\left((\hat{Y}_{p,c})_{i} - (Y_{p,val})_{i} \right)^{2}, $$

where $(\hat {Y}_{p,c})_{i}$ and (*Y*_*p*,*val*_)_*i*_ denote the predicted and true values of the target variable of sample *i* in the validation data set. In case of empty signatures, the prediction $\hat {Y}_{p,c}$ equals the baseline $\hat {\beta }_{0}$ and the MSPE is calculated accordingly. The expectation of *MSPE*_*p*,*c*_ equals the expectation of $(\hat {Y_{c}}-Y)^{2}$ of single future samples. Analysis of the quality of these estimators (MSPE of SC and MC validation data) compared to the true MSPE-value of a signature is shown in Table 2.

**Table 2:** Quality of MSPE-estimation by SC and MC validation data sets

Signature	approx. MSPE	SEM	Validation	estim. MSPE	SEM	squared error	SEM
SC	5.74	0.14	SC	5.49	0.17	15.68	2.04
			MC	5.49	0.12	2.73	0.31
MC	0.87	<0.01	SC	0.87	0.01	0.06	<0.01
			MC	0.88	0.01	0.02	<0.01

The average true MSPE value of a signature discovered in SC or MC data is approximated in 1000 iterations by 10^5^ sample data sets with different random batch patterns on each sample. The approximated MSPE-value is reported with its standard error of the mean as well as the MSPE estimated in the validation data and its standard error. The average squared error of this estimator ((*MSPE*_*estim*_−*MSPE*_*approx*_)^2^) was calculated from 1000 discovery data sets with 100 independent validation data sets each

#### (C) successful validation: SV

The lowest requirement of a signature is a performance on independent validation data significantly better than a random prediction. This is indicated by a positive (significantly larger than zero) correlation between the predicted values of the target variable $\hat {Y}_{p,c}$ and the true values of the target variable *Y*_*p*,*val*_ in the validation data (sample size identical with that of the corresponding discovery data).

SV equals 1 if p (the *p*-value of a one-sided correlation test on $(\hat {Y}_{p,c},Y_{p,val})$) is smaller than 0.05, and 0 otherwise.

For empty signatures, there is no successful validation possible and therefore SV is set to 0.

Note that statistical significance is a problematic performance score. Firstly, in real data applications anything will be significant with sufficient sample size, regardless of the true effect size. Secondly, the goal of clinical biomarkers is not merely to perform slightly better than a random prediction. Nevertheless, at least for candidate screening studies, successful validation is an important milestone.

#### (D) calibration slope: CS

CS is a common measure of prediction quality. It is calculated as the slope in a simple linear model regressing the validation data outcome on the predicted values. $ Y_{p,val} = a + c \cdot \hat {Y}_{p,c} + \epsilon $ with $\epsilon \sim \mathcal {N}(0,\sigma _{c}^{2})$. Let $\hat {c}$ denote the estimated coefficient *c*, then $ CS_{p,c} = \hat {c}$. Since constants are uncorrelated with any data, the CS of empty signatures is set to 0. Note that CS indicates an association of the predicted with the observed data on average, not regarding the variance, and it is precisely this variance that can be very harmful in single sample prognosis.

## Results

In three different simulation scenarios, the factors signal strength, number of informative genes and sample size were analyzed, one at a time. Their influence on the predictive performances achieved, when using different combinations of SC and MC data for discovery and validation, are reported in the following. Data generating code, data files as well as functions for reporting the numbers and creating the figures can be downloaded via this link. 1

### Scenario 1: signal strength

**Fig. 2:**
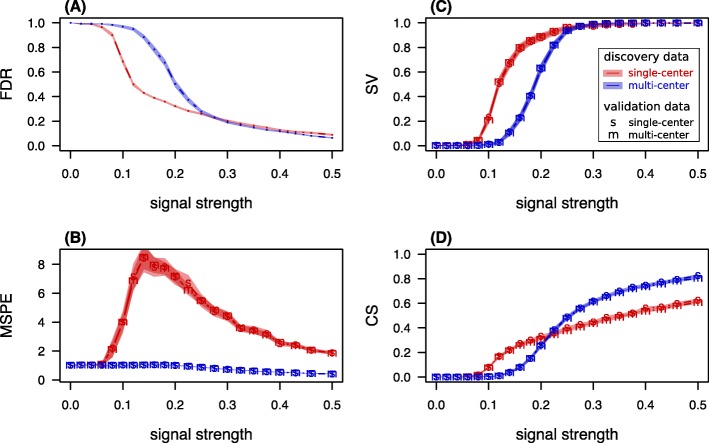
Performance scores under varying signal strength. Performance scores and 99%-confidence bands for **a** “expected fraction of false findings in signature” FDR, **b** “expected error on single future predictions” MSPE, **c** “chance of successful validation" SV, and **d** “average calibration slope” CS calculated from 10^3^ simulation runs. The parameter values are given in Table 1, signal strength is varied in terms of the parameter $\tilde {\beta }$

The true state *a*_*ij*_ affects the raw gene expression data *x*_*ijg*_ of a gene *g* through the parameter $\tilde {\beta }_{g}$. The average parameter value of the informative genes is denoted $\tilde {\beta }$ and called signal strength. In the first scenario, the signal strength was varied systematically from 0 to 0.5, while all other parameters were kept constant at values provided in Table 1. The average performance scores according to simulation scenario 1 are presented in Fig. 2.

For signal strengths of $\tilde {\beta }$ lower than 0.1, the average FDR was higher than 0.9 for both MC and SC discovery data, indicating that the signal was too low for prognostic modeling under the given parameters independently of the data type. For $\tilde {\beta }$ between 0.1 and 0.2, the FDR in SC signatures was significantly lower than the one of MC signatures (e.g. $\tilde {\beta } = 0.14$: mean FDR in SC signatures 0.429±0.003 SEM; mean FDR in MC signatures 0.885±0.008).

At the same $\tilde {\beta }$ of 0.14, the average MSPE of the prognostic models trained on SC data and validated on MC data was 8.51 (±0.32), in contrast to the average of 1.03 (±0.01) in the setting, where MC data was used for discovery and SC data was used for validation.

Thus, the low FDR of SC signatures was accompanied by a high MSPE. This discrepancy in the two scores underlines the multidimensional nature of quality concepts for prognostic models; data usage strategies may perform better with respect to one score but worse with respect to the other. To examine the role of empty candidate signatures in the reported performance scores, we conducted further simulations and analyzed the chance to discover a non-empty candidate signature, the mean signature length as well as the performance scores of the prognostic model, under the exclusion of those cases where no signature was discovered at all (see supplementary information). Heterogeneous batch patterns of MC data, for instance, bury weak signals ($\tilde {\beta } < 0.15$) and thus no informative features enter the signature. At the same time the prediction error stays in the range of random predictions unless the signal is strong enough to systematically reduce this error ($\tilde {\beta } > 0.2$). In contrast, homogeneous batch patterns of SC data sometimes allow identification of informative features, but the predictions are of low accuracy and accompanied by a dramatic increase in the MSPE. Therefore, at $\tilde {\beta } = 0.15$ with respect to quality criterion A, SC discovery was the best choice, while with respect to quality criterion B, MC discovery was the best choice.

We also investigated the dramatic increase of the MSPE of SC signatures, which turned out to be a result of the disturbing effect of the homogeneous batch pattern on the precision of Lasso parameter estimation (data not shown).

The chance to successfully validate a signature was higher for SC than MC discovery for all intermediate signal strengths ($\tilde {\beta }$ between 0.08 and 0.25). This range corresponds to a lower mean FDR and a higher mean MSPE of SC settings, reflecting that the prediction values showed an increased variance but are nonetheless correlated with the true outcome to a verifiable degree. For weaker signals the success rate was zero for all settings, whereas for stronger signals confirmation of association was certain.

The calibration slope was zero in all SC-MC combinations for $\tilde {\beta } < 0.08$. At lower intermediate signals ($\tilde {\beta }$ between 0.1 and 0.2), the average calibration was better for signatures trained on SC data.

For stronger signals ($\tilde {\beta } > 0.3$), MC discovery outperformed SC discovery in all measured scores. Mixed designs (i.e. SC discovery data validated on MC data and vice versa) had consistently similar performance scores as the homogeneous designs when considering the same type of discovery data (see Fig. 2). In other words, with respect to signal strength, there were no notable differences in performance between validation on SC data or MC data, independent of where the signature was discovered.

The quality of the MSPE-estimator itself using SC or MC data is shown in Table 2, where in 1000 iterations, huge data sets of 10^5^ samples were generated for approximation of the true MSPE expectation for future single samples at a signal strength of $\tilde {\beta } = 0.25$. Both SC and MC validation data lead to unbiased estimation of the true MSPE. The average squared error of the estimated MSPE, however, was considerably larger in SC validation data. Results were similar for other scenarios (data not shown). Therefore, MC validation outperformed SC validation considering the same discovery data.

Taking the perspective of an increasing signal strength and focusing on the FDR, it was the SC setting that first dropped, indicating the successful identification of informative genes among the 1000 measured features. The MC setting did not catch up unless the signal reached a strength at which both settings had FDR values lower than 0.25 and showed similar FDR values for further increasing signal strengths. From this point of view, it is always advisable to discover on SC data. But the expected quality of the prediction contradicts this advice. With respect to the MSPE, MC discovery was the dominant strategy and with respect to the calibration slope, SC is only the better strategy for low signals, where the prediction quality was poor anyway. Starting at an effect size of $\tilde {\beta } = 0.225$ the MC setting outperformed the SC setting with respect to all considered performance scores. Interestingly, this turning point coincided with the point for which the MC setting reached an expected success rate of 0.8, which corresponds to the targeted success rate of common sample size calculations.

### Scenario 2: number of informative features

**Fig. 3:**
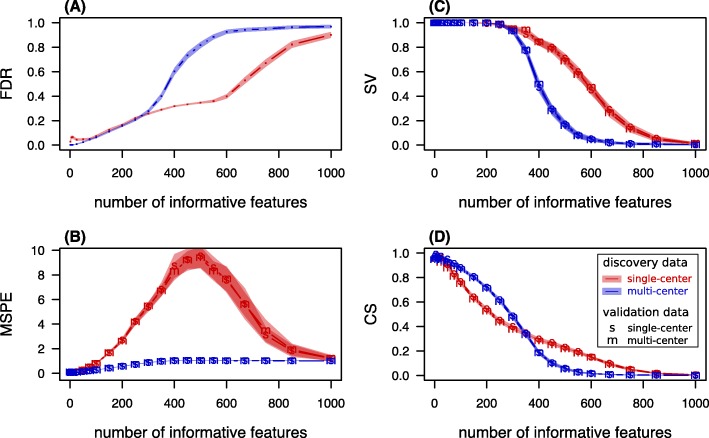
Performance scores under varying number of informative genes. Performance scores and 99%-confidence bands for **a** “expected fraction of false findings in signature" FDR, **b** “expected error on single future predictions” MSPE, **c** “chance of successful validation" SV, and **d** “average calibration slope” CS calculated from 10^3^ simulated prognostic modeling iterations. The parameter values are given in Table 1 and the number of informative genes is varied. Note that the overall signal ($\sum _{g} \tilde {\beta }_{g} \equiv \beta \cdot n_{inf}$) is kept constant by adapting $\tilde {\beta }$ to the number of informative features *n*_*inf*_

In a second scenario, the number of informative features was varied from 1 to 1000, while the total number of features was kept constant at 1000. An informative feature is a gene *g* with a true *β*_*g*_≠0. The signal strength of $\tilde {\beta } = 0.25$ was therefore distributed over varying numbers of informative genes, while the sum of coefficients was kept constant. In the extreme cases, one feature carried the whole signal or the signal was spread over all features. The average performance scores according to simulation scenario 2 are presented in Fig. 3.

For 250 informative features or less, the mean FDR of MC signatures was lower than for SC signatures, with a maximum difference of 0.063±0.005 measured at *n*_*inf*_=10. For 300 or more informative genes, the average FDR was higher for MC signatures than for SC signatures, with a maximum difference of 0.528±0.010 measured at *n*_*inf*_=600. Note that high FDR values also indicate empty signatures, which stem from unsuccessful discovery attempts in spite of existing signal.

Analogously to the first scenario, the most important performance criterion MSPE was higher for the signatures discovered in SC data compared to the MSPE of MC signatures.

Nonetheless, successful validation was more likely when following the strategy associated with the higher MSPE values for a number of informative genes between 350 and 750.

Most interestingly, the mean calibration slope was higher for MC signatures when the information contained in $\tilde {\beta }$ was spread across fewer than 350 features and lower otherwise.

Thus, according to the MSPE, the MC discovery setting was superior. With respect to the other performance criteria at the cost of high MSPE values, there was benefit in SC discovery when the signal was spread over many features. Consistently with the first scenario, the choice of validation data was meaningless apart from the quality of the MSPE estimation.

### Scenario 3: number of samples

**Fig. 4:**
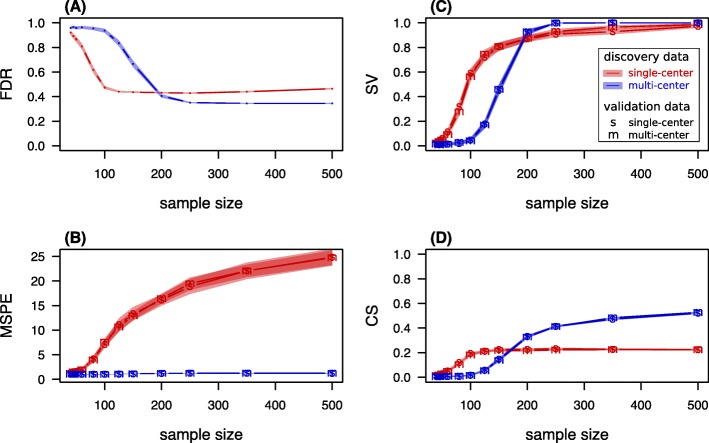
Performance scores under varying sample size. Performance scores and 99%-confidence bands for **a** “expected fraction of false findings in signature" FDR, **b** “expected error on single future predictions" MSPE, **c** “chance of successful validation" SV, and **d** “average calibration slope” CS calculated from 10^3^ simulated prognostic modeling iterations. The parameter values are given in Table 1 and the sample size is varied

In a third scenario, the number of samples was varied. This is the most common and easy to adjust parameter when planning a study. In order to unravel the influence of sample size, a low signal strength of $\tilde {\beta } = 0.125$ was chosen. The average performance scores according to simulation scenario 3 are presented in Fig. 4.

In concordance with the first scenario, the FDR in signatures found in SC data was lower than the FDR of MC signatures when the sample size was low. A sample size of at least 200 was required for this weak signal to obtain mean FDR values that were lower in MC signatures than in SC signatures. The maximum gap between the two settings was observed at 100 samples (SC FDR: 0.47±0.15 standard deviation; MC FDR 0.94±0.20).

Consistently with the previous two scenarios, the SC discovery scenario lead to systematically higher MSPE values and the choice of validation data made no difference. This gap between the MSPE values of single- and MC signatures even grew with sample size, as more and more features entered the signature.

For sample sizes up to 150 samples, the rate of successful validation in SC signatures was higher than for MC signatures. For 200 samples or more, the MC discovery setting showed a higher success rate.

The same tendency was found in the average calibration slope. While for up to 150 samples the SC scenario had higher average slope, the average calibration slope did not increase notably with larger samples sizes (SC discovery, MC validation: 150 samples: CS =0.217±0.003; 500 samples: CS =0.224±0.002). In contrast, in the MC discovery setting the performance increased substantially within this range (MC discovery, SC validation: 150 samples: CS =0.146±0.005; 500 samples: CS =0.521±0.003).

Thus, except for low- to under-powered studies, where SC signatures had a lower FDR, SV and CS, MC settings clearly outperformed the SC approach. In particular, MC discovery had lower expected prediction error than SC discovery, independently of the sample size.

## Discussion

### Summary

In this article, we investigate how to make best use of SC and MC gene expression data sets for prognostic modeling, where our results are particularly relevant for the prediction of therapeutic success of radiotherapy. To mimic the process of discovery and validation of molecular signatures, we generated in silico high-dimensional continuous data carrying signal, white noise and center specific noise patterns. The computer generated data sets were then processed in an analysis flow that would also be applicable to gene expression data in everyday experiments: First, ComBat batch correction was applied, subsequently prognostic models were built using Lasso followed by determining the performance using various scores.

Comparing the quality of two prognostic models is a complex task, since quality refers to many different aspects of a model and hence is a multidimensional concept. For quality measures of clinical trials, which face the same difficulty, it is known that the use of summary scores is problematic [43]. In particular, a model should be evaluated in reference to the task for which it was designed [29]. Overviews of available scores and their applications can be found in various articles [29, 44–48]. These performance scores all have in common that the performance of a signature is evaluated in light of new data with known outcomes. When variable selection steps are implemented in the model fitting procedure, their quality can be addressed separately as well [30]. Consequently, in our analyses for evaluating the quality of the choice of data usage in molecular prognostic modeling, four performance scores are used, where each of these addresses a specific aspect of success. Interestingly, there is no dominant strategy in terms of one type of data outperforming the other independently of the signal strength and sample size.

To understand why the lines of performance scores (FDR, SV, CS) cross for MC and SC discovery, consider the following two situations: First, if the signal strength or the sample size is low or if the signal is diffused by being distributed over many variables (high number of informative features), the signal in MC discovery data gets lost among the various batch patterns of the different parts constituting this data. In this situation, using SC discovery data can be advantageous, because the signal detection on the discovery data works better as the whole discovery data set shares the same batch effect pattern for SC discovery. Second, in contrast, if the signal is strong, the sample size large, or if the signal concentrates in few variables with strong influences (small number of informative features), MC discovery data is advantageous. In the just described situations the signal in MC discovery data is no longer buried among the various batch patterns associated with MC data. The fact that the SC data carries only a single batch effect pattern is a disadvantage of SC discovery in these situations, because the resulting signature is overly well adjusted to the discovery data associated with this specific batch effect pattern.

The results of our simulation study show that the decision on which data to use for discovery of a signature is connected with the intention of the study the molecular prognostic model is built for. If the study is designed to produce a gene signature that can be instantly applied to decision making about radiotherapeutic options, the focus lies on the minimization of the expected prediction error and thus the precise parameter estimation of the signature coefficients. In this case, FDR, SV and CS are not the parameters of interest and therefore our simulation results clearly advise the use of MC discovery data and, if available, the use of MC data also for validation.

In contrast, if the study is designed to identify candidate biomarkers in an exploratory project, the focus lies on the successful identification of information carrying genes. In this case FDR and SV are the performance scores of interest rather than minimizing the MSPE. Therefore our simulation results indicate advantages of SC discovery over MC discovery, if the anticipated signal-to-noise ratio and the sample size are small. Again, it is advisable to aim for MC validation data.

This dependency of the data usage advice on the intention of the study nicely fits into the scene presented by Altman and Royston, who examined the complexity of the validity concept for prognostic models and its dependence on the model purpose, which is reflected in a context-dependent definition of performance adequacy [29].

### Limitations of the study

In general, the partial lack of transferability of a simulation study limits its benefit. Four limitations of our study design are discussed in the following, to make the scope of our results more transparent.

First of all, we use a microarray batch effect model to mimic variation between centers and batch effects generally characterize biases of separately generated parts of the same data. If this model captures center effects in gene expression data of tumor tissue insufficiently, all findings only apply to studies affected by batch effects rather than center effects. To critically assess this assumption of center heterogeneity being adequately modelled by the Hornung batch model, two questions need to be answered: (I) “Does the Hornung model generate heterogeneity patterns as found in gene expression data from different clinical sites?" and (II) “Are all aspects of heterogeneity of clinical sites covered by the Hornung model?"

With regard to question (I), microarray samples of the same center share the specific tissue sampling procedure that unavoidably introduces variation between centers beyond all standard operating protocols [38]. It has been reported that the list of center-specific factors that affect microarray data is surprisingly long [32–34]. All center-wise factors that introduce shared errors in terms of mean shifts, correlations or scaling effects are captured by the Hornung model. Therefore, we argue that the batch model of Hornung et al. [39] adequately generates heterogeneity characteristics as expected in MC microarray data.

With regard to question (II), we underline that there are further sources of heterogeneity, which we did not model explicitly. Certainly, case mix and varying signal strength are two factors that introduce further heterogeneity between centers that is not included in the noise-terms of the Hornung model. In order to mimic these effects, either the signal vector or distribution parameters (e.g., the standard deviation of normally distributed model parameters) must be varied between centers, thereby implicitly enlarging the list of assumptions and parameters substantially.

Thus, we argue that the Hornung model generates heterogeneity patterns that are well suited to study the performance of prognostic models using populations of patients that are homogenous with respect to biology.

Second, we discuss the performance of a multiple regression model with a continuous response variable instead of censored survival data. Most prognostic models in cancer research are Cox proportional hazard models or, in the case of binary response variables, logistic regression models. Prediction of a continuous outcome from microarray data is rarely seen in practice. Yet, there are established linear predictive models in radiation oncology. For example, the so-called "radiation sensitivity index" successfully predicts tumor radiosensitivity in breast cancer [49]. However, the conclusions drawn from the simulation study can be transferred to types of response variable other than continuous outcomes. The structure of the center effects does not depend on the type of the response variable. Moreover, there seems to be no plausible reason why the general procedures of ‘variable selection’/‘parameter estimation’ and ‘prediction’ would be influenced differently for different types of response variables by the general factors ‘signal strength’, ‘number of informative genes’, and ‘sample size’ [50]. Linear regression is a very basic model and was thus a suitable choice for the simulation study in order to not complicate its design unnecessarily. Nevertheless, there are complex structural differences in MC studies with different types of response variables. For example, in MC studies that feature binary response variables and use logistic regression, marginal effects and effects conditional on center are not the same; for methodological work on logistic regression in MC studies with binary response variables, see Wynants et al. (2018) and Meisner et al. (2019) who include the center effect in the model equations instead of applying batch correction to the MC data beforehand [51, 52].

Third, the choice of scenarios and chosen parameters shapes the outcome of the analysis. Therefore, we took as many parameters as possible directly from Hornung et al. [39] and adapted sample size and number of centers to a real MC data set recently used for a genomic prognostic model [10]. The measurement error of the true state was kept at 10% of the variation over all samples. The remaining parameters were standard normally distributed and thus did not add so-called researcher degrees of freedom, which refers to the many choices researchers have to make during data analysis, thereby increasing the risk of finding over-specific and irrelevant or at worst even false positive results [53]. The choices of the model parameters signal strength, spreading of the signal over many genes as well as the considered sample size were based on our experiences working with real gene expression data sets. There is no doubt that various other settings would also have been of interest.

Fourth, we did not use real tumor data to validate the results from our simulation study. Simulated effect curves gain persuasive power when data points obtained from real data examples are added to the trajectories and match the simulation results. However, the result obtained with a real data example is but a single point in the space of the possible results, which does not allow to draw conclusions on further points (i.e. on the results one would obtain with other data sets). Single observations are expected to differ from the presented lines; they never contradict expected mean values without considering the variance. Furthermore, the true state (i.e. corresponding set of parameter values for the Hornung model) is not known in real tumor data, which is why all model parameters have to be estimated, particularly the signal strength and the number of informative genes. These estimated parameter values can be unreliable or biased and therefore the coordinate of a real data performance score in the presented plots is associated with large uncertainty.

To sum up, given the generality of the simulation design, we are confident that the presented effects are widely applicable to molecular prognostic modeling in various disciplines. To our knowledge, there are no studies that analyzed the performance of feature selection and prognostic modeling approaches in MC settings.

## Conclusion

The simulations clearly show that decision making regarding the choice of multi-center or single-center data for prognostic modeling must consider the study aim and thus the performance criterion of interest. If the study is designed to build a prognostic model for direct application to radiotherapeutic decision making, minimization of the prediction error will have highest priority and thus we recommend use of multi-center discovery data. In contrast, if the study is designed to identify informative genes for future investigations, minimization of the false discovery rate and maximization of the chance of successful validation will have highest priority and thus we recommend use of single-center discovery, if the anticipated signal-to-noise ratio and the sample size are small. Even though multi-center validation data returns better estimates for the true prediction error, we consider this aspect less important than the effect of the choice of the discovery data on the signature’s performance. This simple decision rule may support anybody involved in study design regarding data usage for genomic prognostic models.

## Supplementary information

**Additional file 1** Supplementary simulations. Further plots of the three simulation scenarios, adding “frequency of non-empty signatures”, “mean signature length” and performance scores free from empty signatures (i.e., performance of data usage setting, given a candidate signature was discovered.

## Data Availability

The datasets generated and analyzed during the current study are available at https://www.helmholtz-muenchen.de/fileadmin/ZYTO/other/onlMatSamaga.zip

## Footnotes

1 https://www.helmholtz-muenchen.de/fileadmin/ZYTO/other/onlMatSamaga.zip
