# Supplementary material for: Single-center versus multi-center data sets for molecular prognostic modeling: a simulation study
Source: Radiat Oncol. 2020 May 14;15:109. doi: 10.1186/s13014-020-01543-1 (PMC7227093; doi:10.1186/s13014-020-01543-1)
Supplement: Supplementary file 1 — Additional file 1 Supplementary simulations. Further plots of the three simulation scenarios, adding “frequency of non-empty signatures”, “mean signature length” and performance scores free from empty signatures (i.e., performance of data usage setting, given a candidate signature was discovered. [file 13014_2020_1543_MOESM1_ESM.pdf]

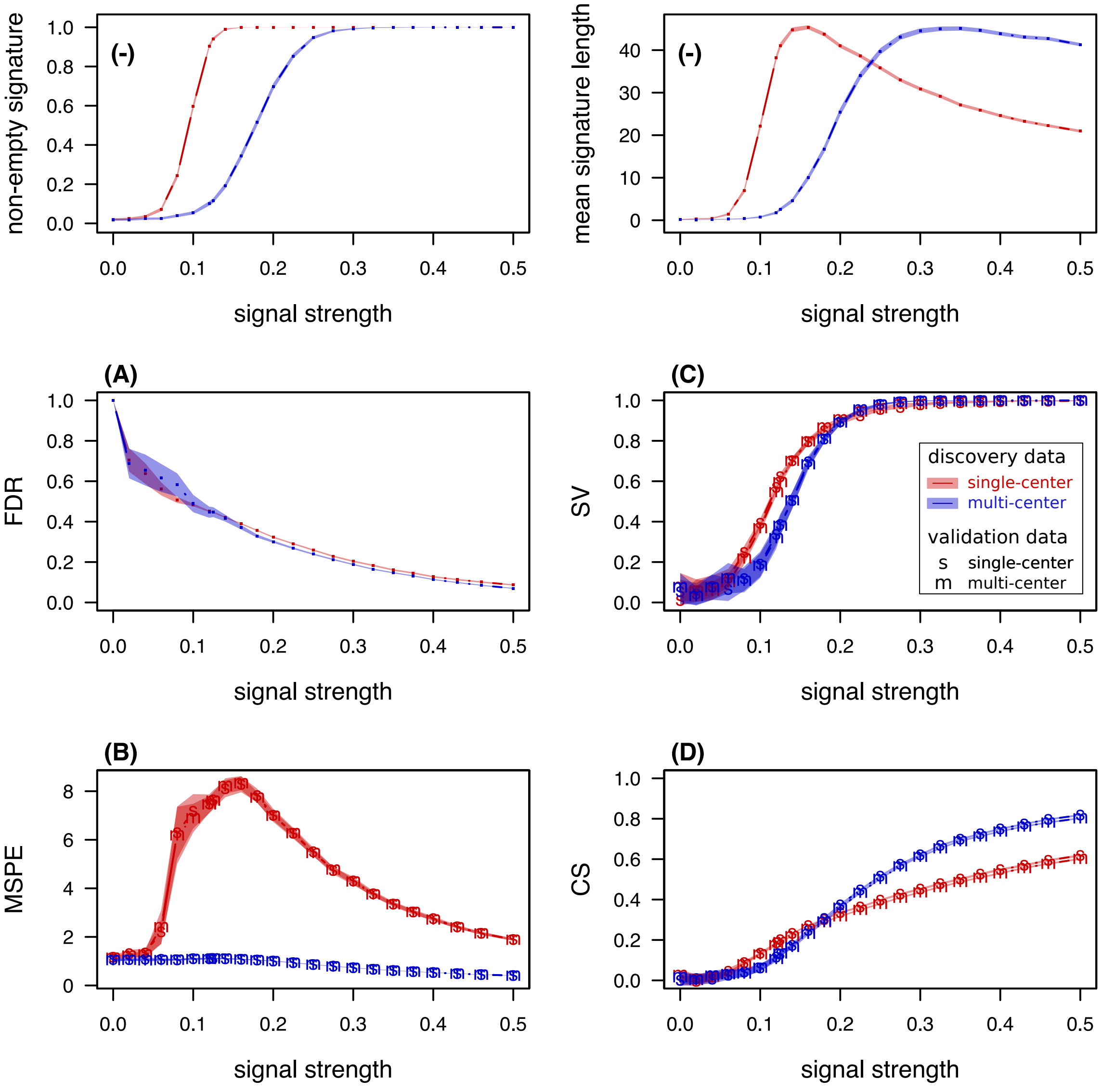

Figure S1: Performance of candidate signatures for scenario (i). Empty signatures are excluded from averaging. Each data point is the result of 5000 simulation runs. The parameter values are given in Table 1, signal strength is varied in terms of the parameter $\tilde{\beta}$. **top left:** fraction of non-empty candidate signatures. **top right:** average number of features in the candidate signature. (A) average false discovery rate (FDR) in non-empty signatures. (B) average mean squared prediction error (MSPE) in non-empty signatures. (C) fraction of successful validations (SV) of non-empty signatures. (D) average calibration slope (CS) of non-empty signatures. Shaded areas indicate 2.58-fold standard error of the mean, and thereby approximate the 99%-confidence intervals of the mean.

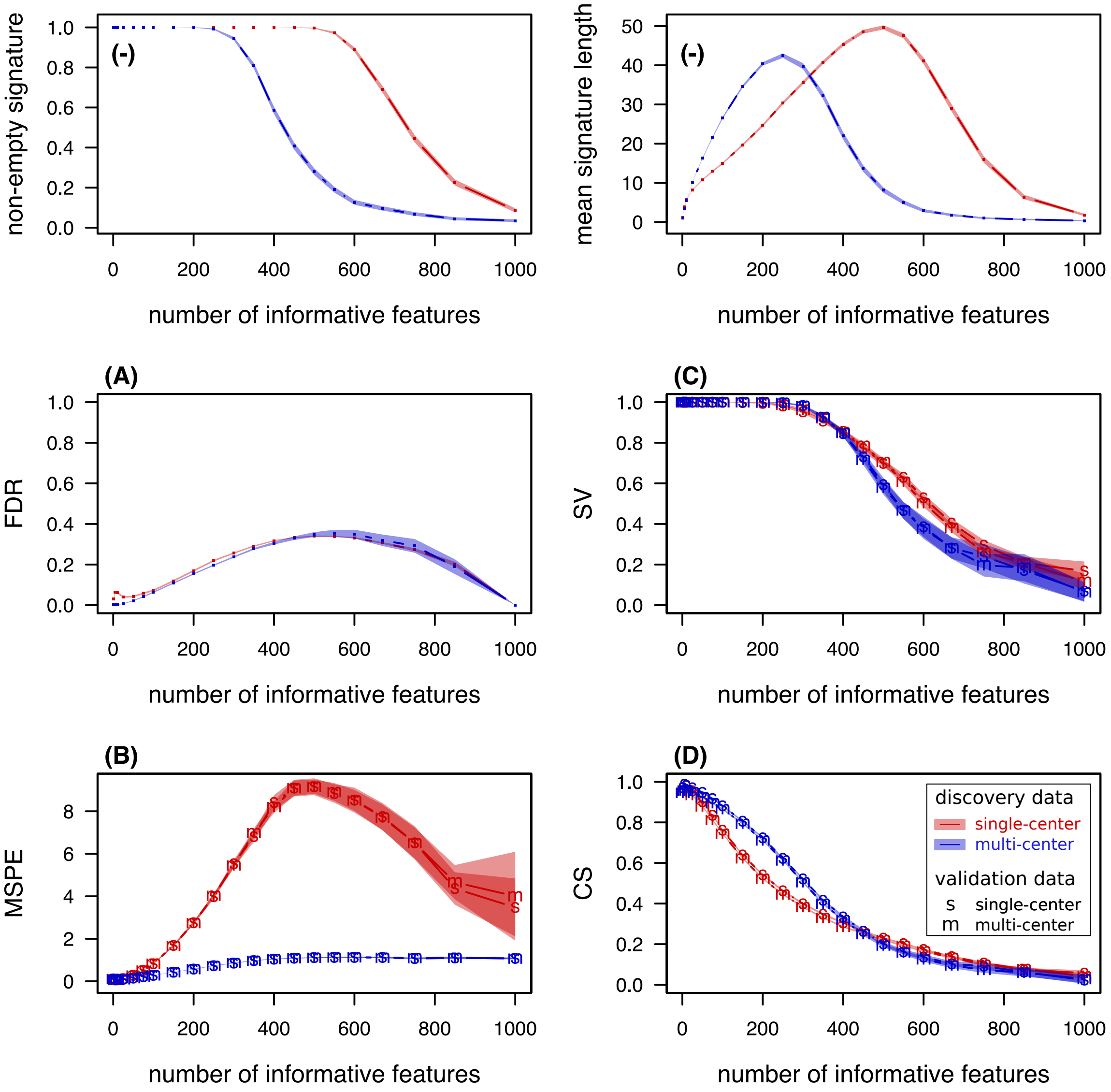

Figure S2: Performance of candidate signatures for scenario (ii). Empty signatures are excluded from averaging. Each data point is the result of 5000 simulation runs. The parameter values are given in Table 1, the number of informative genes is varied. Note that the overall signal $(\sum_g \tilde{\beta} \equiv \beta \cdot n_{inf})$ is kept constant by adapting $\tilde{\beta}$ to the number of informative features n_{inf}. **top left:** fraction of non-empty candidate signatures. **top right:** average number of features in the candidate signature. (A) average false discovery rate (FDR) in non-empty candidate signatures. (B) average mean squared prediction error (MSPE) in non-empty signatures. (C) fraction of successful validations (SV) of non-empty signatures. (D) average calibration slope (CS) of non-empty signatures. Shaded areas indicate 2.58-fold standard error of the mean, and thereby approximate the 99%-confidence bands of the mean.

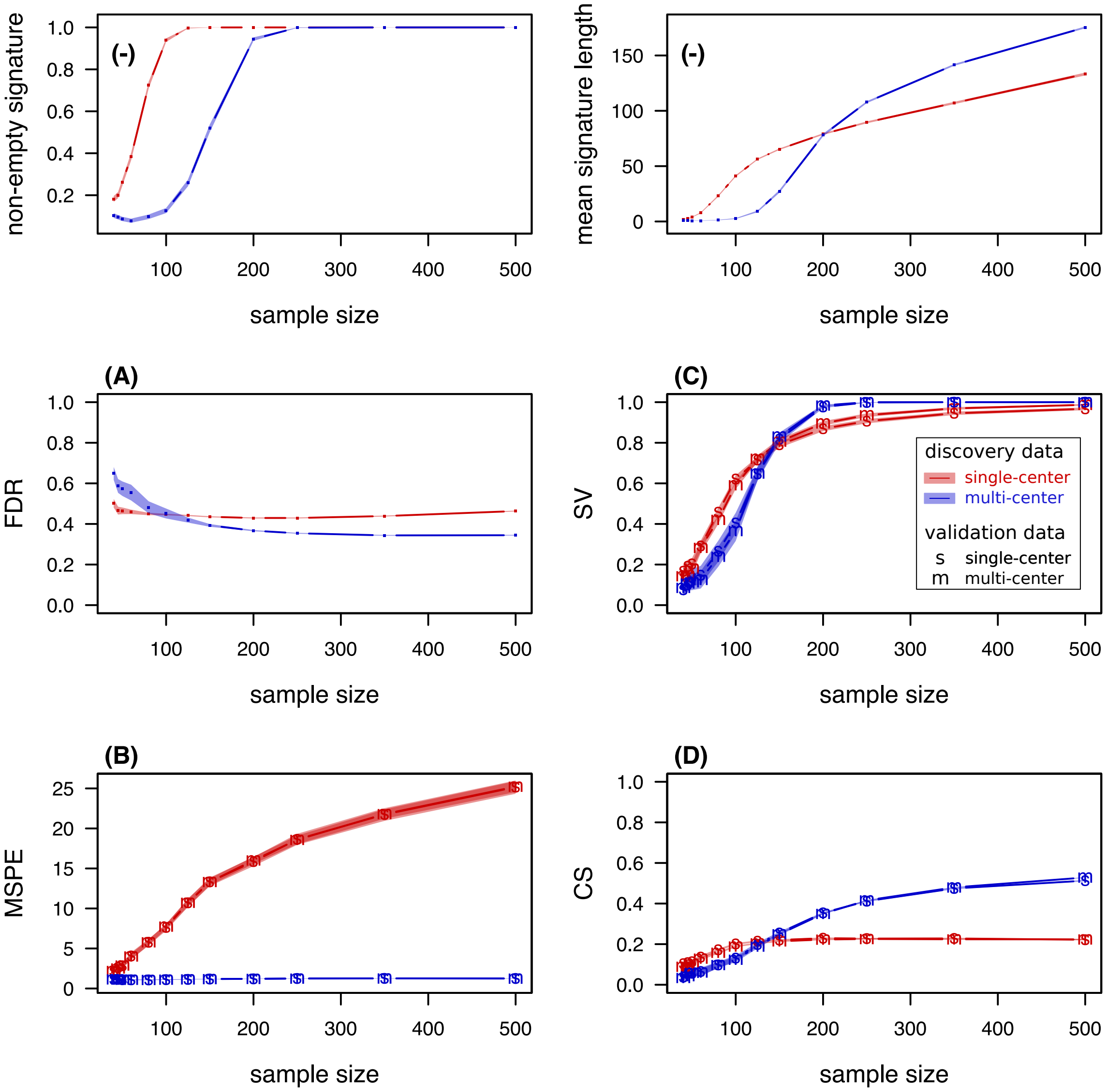

Figure S3: Performance of candidate signatures for scenario (iii). Empty signatures are excluded from averaging. Each data point is the result of 5000 simulation runs. The parameter values are given in Table 1, the sample size is varied. **top left:** fraction of non-empty candidate signatures. **top right:** average number of features in the candidate signature. (A) average false discovery rate (FDR) in non-empty candidate signatures. (B) average mean squared prediction error (MSPE) in non-empty signatures. (C) fraction of successful validations (SV) of non-empty signatures. (D) average calibration slope (CS) of non-empty signatures. Shaded areas indicate 2.58-fold standard error of the mean, and thereby approximate the 99%-confidence bands of the mean.
